# New Personal Model for Forecasting the Outcome of Patients with Histological Grade III-IV Colorectal Cancer Based on Regional Lymph Nodes

**DOI:** 10.1155/2023/6980548

**Published:** 2023-02-25

**Authors:** Jun Yang, Wei Jin, Zhuoqun Lin, Shaotang Li

**Affiliations:** Department of Colorectal and Anal Surgery, The First Affiliated Hospital of Wenzhou Medical University, Wenzhou 325000, China

## Abstract

**Background:**

Metastases at regional lymph nodes could easily occur in patients with high-histological-grade colorectal cancer (CRC). However, few models were built on the basis of lymph nodes to predict the outcome of patients with histological grades III-IV CRC.

**Methods:**

Data in the Surveillance, Epidemiology, and End Results databases were used. Univariate and multivariate analyses were performed. A personalized prediction model was built in accordance with the results of the analyses. A nomogram was tested in two datasets and assessed using a calibration curve, a consistency index (C-index), and an area under the curve (AUC).

**Results:**

A total of 14,039 cases were obtained from the database. They were separated into two groups (9828 cases for constructing the model and 4211 cases for validation). Logistic and Cox regression analyses were then conducted. Factors such as log odds of positive lymph nodes (LODDS) were utilized. Then, a personalized prediction model was established. The C-index in the construction and validation groups was 0.770. The 1-, 3-, and 5-year AUCs were 0793, 0.828, and 0.830 in the construction group, respectively, and 0.796, 0.833, and 0.832 in the validation group, respectively. The calibration curves showed well consistency in the 1-, 3- and 5-year OS between prediction and reality in both groups.

**Conclusion:**

The nomogram built based on LODDS exhibited considerable reliability and accuracy.

## 1. Introduction

Colorectal cancer (CRC) is known as having the third highest incidence, regardless of gender [1], and the predominant type is adenocarcinoma [2]. The situation of cancer is an influencing factor for the outcome of patients with CRC [3–5]. Several studies reported that patients with CRC of different histological grades showed different outcomes [6–10]. High histological grades increased the likelihood of bowel obstruction before surgery [11]. Moreover, metastases at regional lymph nodes could more easily occur in patients with CRC with a higher histological grade [12]. The prognosis could also depend on the condition of regional lymph nodes [5]. Therefore, to ensure lymph node dissection has the desired effect, at least 12 regional lymph nodes should be obtained during surgery, according to the guidelines [13].

In the tumor (T), node (N), and metastasis (M) staging system of the American Joint Committee on Cancer (AJCC), pathologically positive regional lymph nodes were used as a criterion for stratifying patients [5]. The indicators involved in this system are intuitive and easy to obtain. However, the number of examined regional lymph nodes (ELNs) in the N stage was not considered. Hence, the lymph node ratio (LNR) was proposed as a supplement to the N staging system [14].

LNR refers to the proportion of positive regional lymph nodes (PLNs) in ELNs. It was reported to be a good predictor of outcomes in several kinds of cancer [15–18]. What's more, a study focused on right colon cancer pointed out that LNR was a potentially valuable factor in predicting the chance of tumor recurrence [15]. However, when the number of PLNs is 0, the value of LNR does not change regardless of the number of ELNs. Log odds of positive lymph nodes (LODDS), which refers to the logarithm of result of the PLNs divided by negative lymph nodes (NLNs), showed better prediction ability than LNR in several studies [19–21]. LODDS could make up for the deficiency in LNR. Nowadays, few tools built based on LNR or LODDS could be used to evaluate the overall survival (OS) of patients with poorly differentiated or undifferentiated CRC, even though metastases at regional lymph nodes could easily occur in these patients [12].

By utilizing the Surveillance, Epidemiology, and End Results (SEER) database [22], the prognostic factors for patients with histological grades III-IV CRC were explored on the basis of clinical factors, including LNR and LODDS. A personalized prediction model that could be used to make clinical decisions was further constructed and validated.

## 2. Method

### 2.1. Data Sources

Cases in the SEER database were used in this study (November 2020 Submission deltails could be acquire at the website: https://seer.cancer.gov/data-software/documentation/seerstat/nov2020/). In this database, the sources of the cases cover all 18 states in the USA [22].

### 2.2. Included Participants

The inclusion criteria were as follows: (1) patients aged 18–80 years; (2) diagnosis was confirmed by positive histology; (3) clinical and follow-up data of patients were completed and available; (4) poorly differentiated or undifferentiated CRC with histological grade III-IV; (5) patients with one primary cancer only. The exclusion criteria were as follows: (1) patients with an autopsy or death certificate only; (2) patients whose overall survival times were less than 1 month; (3) patients with two or more primary cancers. Multiple primary cancer refers to cancer with a site and histological type different from those of first primary cancer, according to a previous study [23].

### 2.3. Variates and Definitions

In this study, demographic information (age and gender) and the characteristics of cancer (primary location, histological type, histological grade, AJCC TNM stage, LNR, and LODDS) were considered. Age was categorized into three levels following a previous study [24]: <45, 45–60, and >60 years. The information from the primary site was recoded on the basis of the second edition of the International Classification of Diseases for Oncology (ICD‐O‐2). The primary site was divided into the right colon (from the cecum to the transverse colon, but the appendix was excluded), the left colon (from the splenic flexure, descending to the sigmoid colon), and the rectum (rectosigmoid junction and rectum). Histologic codes 8140–8389 were identified as adenocarcinoma, 8480–8481 were defined as mucinous adenocarcinoma/mucin-producing adenocarcinoma (AM/MPA), and 8490 were defined as signet ring cell carcinoma (SRCC). The histologic codes were coded on the basis of ICD‐O‐2. Poorly differentiated cancer was defined as histological grade III, and undifferentiated cancer was defined as histological grade IV. NLNs were calculated using the following formula: NLNs = ELNs − PLNs. The value of LNR in every case was calculated in accordance with the formula LNR = PLNs/ELNs [15–18]. The value of LODDS in every case was calculated as follows: LODDS = log ((PLNs + 0.5)/(NLNs + 0.5)) [20]. The cutoff values of LNR, ELNs, and NLNs were decided on the basis of the Kaplan–Meier method. On the basis of these cutoff values, LNR, ELNs, and NLNs were divided into two subgroups. LODDS was divided into three levels following Lee et al. [25]: <−1.3222, from −1.3222 to −0.5863, and >−0.5863. Survival months were calculated as survival months = FLOOR ((endpoint − date)/days in a month)), as defined in the SEER database (details could be acquired at website: https://seer.cancer.gov/survivaltime). OS refers to the time from the day of diagnosis to the day of death.

### 2.4. Risk Factors

A seven-to-three ratio was used to randomly divide all cases into construction and validation groups. The cases in the two groups were then compared. The mean and standard deviation (SD) were used to describe the continuous variables. Logistic regression analyses were conducted sequentially for the initial screening of risk factors associated with patients' OS [26], and the least absolute shrinkage and selection operator (LASSO) regression algorithms were utilized. A cross-validation was also performed to explore the optimal tuning parameters (*λ*), and the most significant variables were screened out. Moreover, a 95% confidence interval (CI) and odds ratio (OR) were used to quantify the effect of features on OS. Then, a generalized linear model was constructed. A forest plot was drawn to display the model visually. The receiver operating characteristic curve (ROC) and area under the curve (AUC) were obtained in the construction and validation groups to evaluate the model's predictive accuracy. The AUC values ranged from 0.5 to 1.0; the larger the AUC, the more reliable the model. Cox regression analyses were performed subsequently [26]. The hazard ratio (HR) and its 95% CI were applied to quantify the results. Schoenfeld's global test [27] was used to verify whether the variables conformed to the proportional hazard (PH) assumption. Deviance residual diagrams were used to evaluate the distribution of data in each variable.

### 2.5. Nomogram Construction and Validation

By referring to the results of the above analyses, a nomogram was developed. Nomogram is known as a reliable tool to predict prognosis, and it displays risk factors visually. The concordance index (C-index) was separately calculated in the two datasets. Furthermore, 1-, 3-, and 5-year ROC analyses were performed, and AUCs were calculated to assess the nomogram's predictive accuracy. The calibration curves in the two groups were obtained via 1000 resamples bootstrapping method to test the consistency between the prediction of the established model and reality.

### 2.6. Statistical Analysis

SEER*∗*Stat (version 8.4.0) was used to collect data. Categorical variables were coded numerically and tested using the chi-square test or Fisher exact test, while continuous variables were tested using ANOVA to describe the characteristics between the two groups. Logistic and Cox regression analyses were conducted for variable selection [26]. The C-index, ROC, AUC, and calibration curves in the two groups were calculated or plotted. All the analyses and figures were performed or plotted using R software (version 4.1.2, https://www.r-project.org/). Packages such as “survival,” “survminer,” “caret,” “tableone,” “glmnet,” “forestplot,” “pROC,” “ezcox,” and “timeROC,” were used in this study. *P* values (two-sided) = 0.05 were considered statistically significant.

## 3. Results

### 3.1. Characteristics of Patients Identified

**Figure 1:**
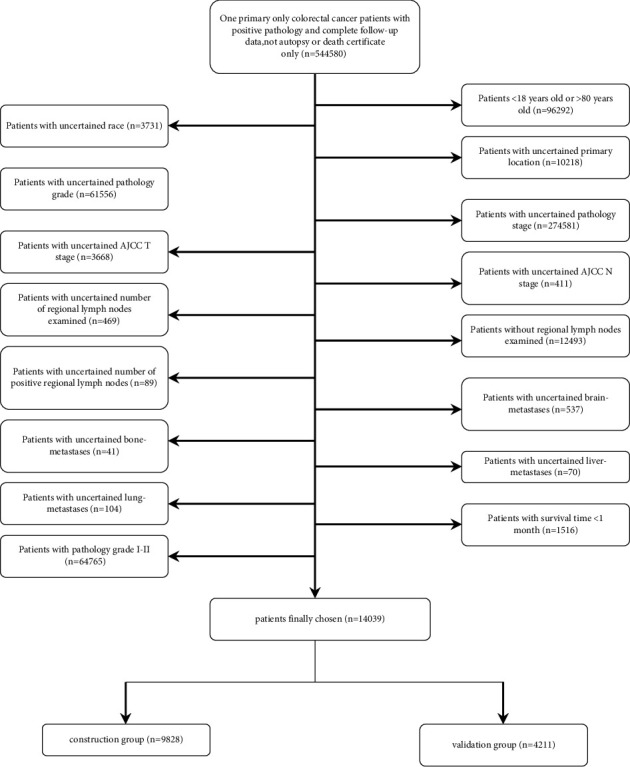
The workflow of the patients chosen.

A total of 14,039 cases were downloaded from the SEER database and divided randomly into construction (9828 cases) and validation (4211 cases) groups. The process of patient selection is shown in Figure 1.

**Figure 2:**
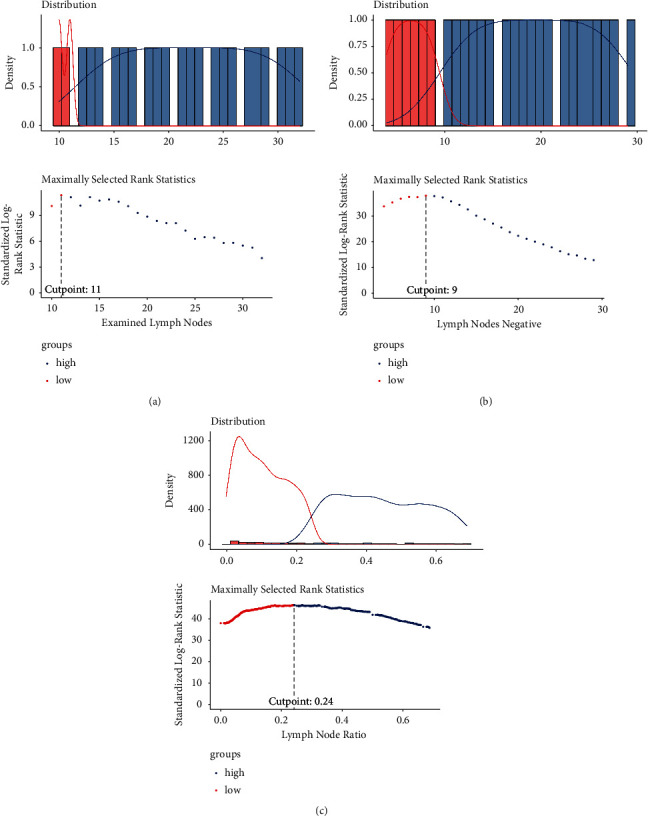
The cutoff values of examined and negative regional lymph nodes and lymph node ratios. (a) The cutoff value of examined regional lymph nodes; (b) the cutoff value of negative regional lymph nodes; (c) the cutoff value of the lymph node ratio.

The cutoff values of LNR, ELNs, and NLNs were calculated separately, as shown in Figures 2(a)–2(c).

The patients were separated into two subgroups in accordance with their LNR status as low (≤0.24) and high (>0.24), their ELN status as low (≤11) and high (>11), and their NLN status as low (≤9) and high (>9), respectively. The characteristics of the cases in the two groups are listed in Table 1. More than half of them were older than 60 years (7839 cases, 55.8%); male (7167 cases, 51.1%); at the AJCC T3 stage (7775 cases, 55.4%); and located in the right colon (7645 cases, 54.5%). Most of them were white people (11,219 cases, 79.9%); at the AJCC M0 stage (10761 cases, 76.7%); grade III (11,721 cases, 84.5%); LNR ≤0.24 (9507 cases, 67.7%); without bone metastases (13,938 cases, 99.3%); without brain metastasis (14,004 cases, 99.8%); without liver metastasis (11,953 cases, 85.1%); without lung metastasis (13,913 cases, 97.0%); high ELNs (12,563 cases, 89.5%); and high NLNs (10,810 cases, 77.0%). The pathological tissue type with the largest proportion was adenocarcinoma (12,112 cases, 84.5%). A total of 5418 cases (38.6%) resulted in death, while 8621 (61.45%) cases were alive in this study. The survival time was 33.28 months (SD = 22.82 months) in total, with33.49 months (SD = 22.95 months) in the construction group and 32.80 months (SD = 22.51 months) in the validation group. No statistical difference was found among all variables between the two groups.

### 3.2. Exploration of Factors for Patients with Histological Grade III-IV CRC

**Figure 3:**
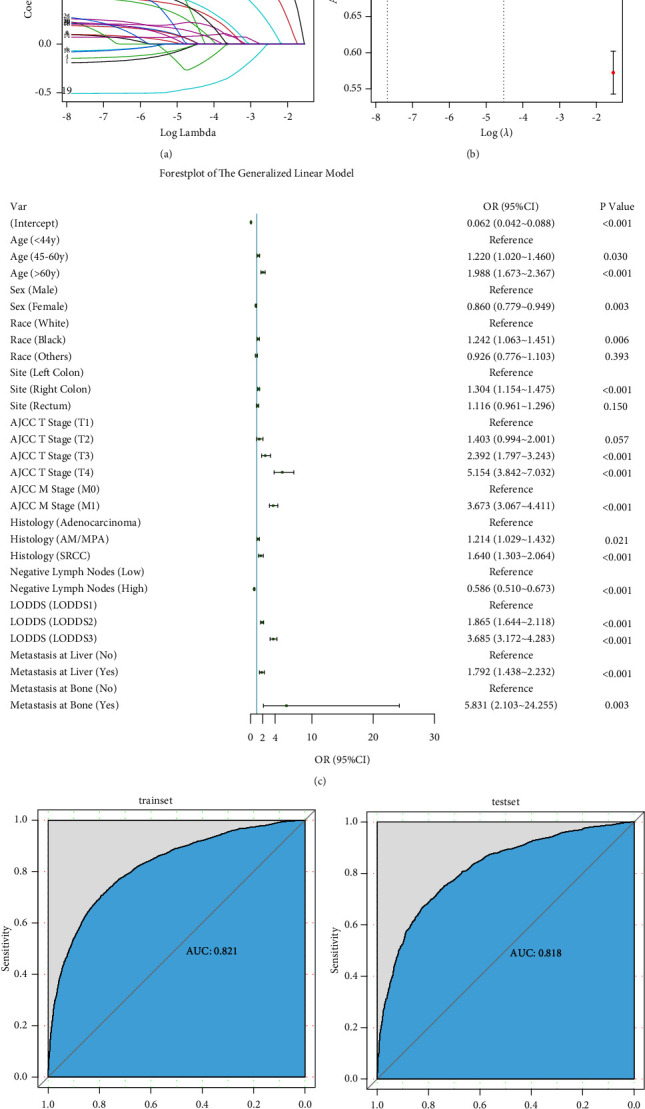
Multivariate logistic regression analysis. (a) LASSO coefficients of features; (b) selection of tuning parameter (*λ*) for LASSO model; (c) forest plot of generalized linear model; (d) receiver operating characteristic curve (ROC) analysis and the area under the curve (AUC) in a training cohort; (e) receiver operating characteristic curve (ROC) analysis and the area under the curve (AUC) in a testing cohort. Site means the primary site of colorectal cancer.

The univariate and multivariate analyses were first performed in logistic regression to initially screen the risk variables for patients with histological grades III-IV CRC. The LASSO regression algorithm was used in this process. According to the multivariate logistic regression analysis (Lambda.1SE = 0.01087546, Figures 3(a) and 3(b)), variables including age, sex, race, a primary site of cancer, histological grade, T, N, M, histological type, LNR, LODDS, NLN, bone metastasis, and lung metastasis were screened out (*P*  <  0.05).

**Figure 4:**
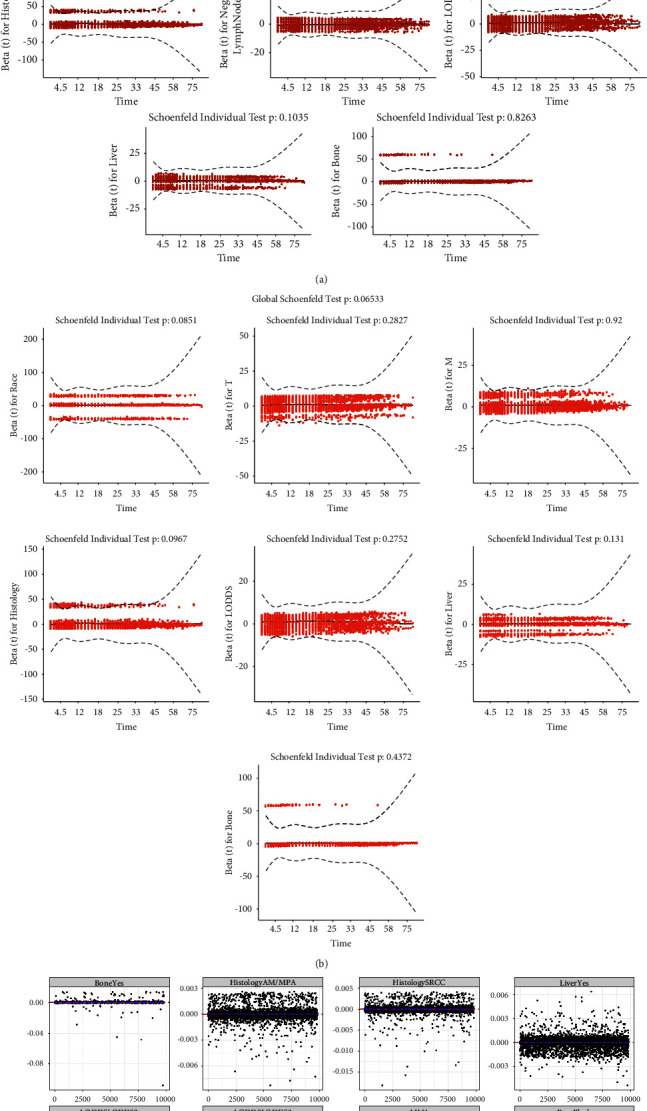
The result of Schoenfeld's global test and deviance residual diagram. (a) The result of Schoenfeld's global test is based on all variates screened out by univariate Cox regression analysis. (b) The result of Schoenfeld's global test after the exclusion of variates that do not conform to the proportional hazard test. (c) The deviance residual diagram indicated the residuals of the variates involved in the nomogram were in a symmetric pattern and had a constant, uniform spread throughout the fit.

The detailed results of the logistic regression analyses are shown in Table 2. Patients aged 45–60 and beyond 60 years, female, black, right colon, T3 stage, T4, M1, AM/MPA, SRCC, LODDS from −1.3222 to −0.5863, LODDS ≥−0.5863, high NLN, bone metastasis, and liver metastasis were preliminarily identified (*P*  <  0.05). Subsequently, a generalized linear model was built, as shown in Figure 3(c). The ROCs were drawn, and the corresponding AUC values were calculated to assess the reliability of the established model, as shown in Figures 3(d) and 3(e). The AUC values in the construction and validation groups were 0.821 and 0.818, respectively, indicating that the established model had a high degree of predictive capacity. The Cox regression analyses were performed for further exploration (Table 3). Schoenfeld's global test was also conducted, and the results are shown in Figures 4(a) and 4(b).

Age, sex, primary site, and NLN did not conform to the PH assumption (*P*  <  0.05) and were thus excluded from the following analyses. The remaining variables, including race, T, M, histological type, LODDS, liver metastasis, and bone metastasis, conformed to the PH assumption (*P*  <  0.05). The deviance residual diagram in Figure 4(c) indicated that the residuals of all variables involved in the nomogram were in a symmetric pattern and had a constant, uniform spread throughout the fit. The results of multivariate Cox regression analysis showed that black race, T3, T4, M1, SRCC, LODDS from −1.3222 to −0.5863, LODDS ≥−0.5863, NLN, metastasis at the bone, and metastasis at the liver, resulted in a worse outcome, whereas other race patients led to an enhanced outcome (*P*  <  0.05).

### 3.3. Construction and Verification of Nomogram

**Figure 5:**
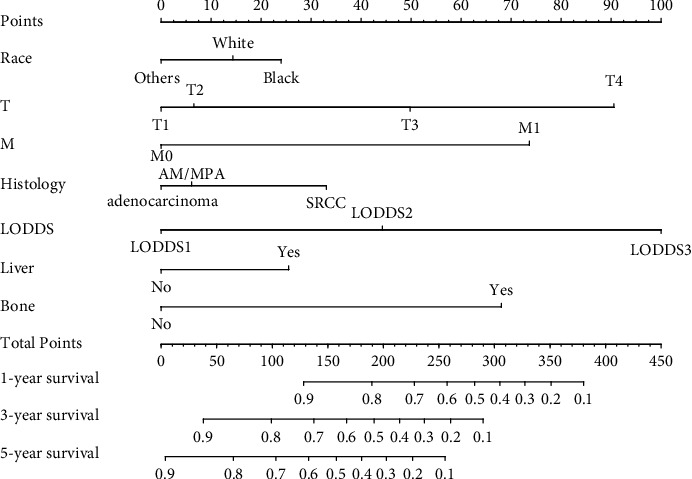
Prognostic nomogram of colorectal cancer patients with grades III-IV based on 7 risk factors.

A nomogram was constructed, as shown in Figure 5.

**Figure 6:**
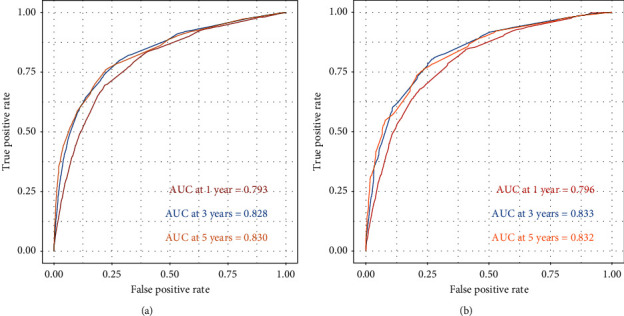
1-year, 3-years, and 5-year receiver operating characteristic curve analysis and the area under the curve in (a) the construction cohort and (b) the validation cohort.

The C-index of this nomogram in the construction and validation groups was 0.770. The results of 1-, 3-, and 5-year ROC analyses in the construction and validation groups are displayed in Figures 6(a) and 6(b).

**Figure 7:**
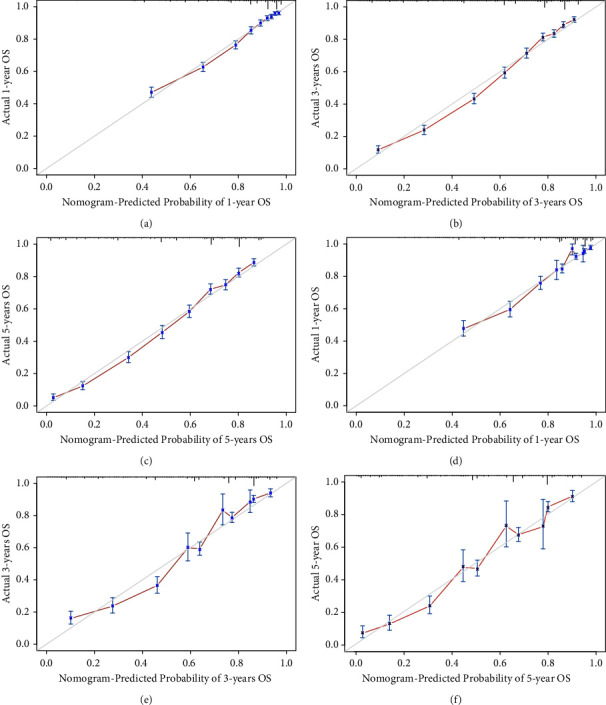
Overall survival calibration curves in the construction cohort and validation cohort. (a) 1-year OS calibration curve in the construction cohort; (b) 3-years OS calibration curve in the construction cohort; (c) 5-years OS calibration curve in the construction cohort; (d) 1-year OS calibration curve in the validation cohort; (e) 3-years OS calibration curve in the validation cohort; (f) 5-years OS calibration curve in the construction cohort.

The 1-, 3-, and 5-year AUC values in the construction group were 0793, 0.828, and 0.830, respectively, and those in the validation group were 0.796, 0.833, and 0.832, respectively. The 1-, 3-, and 5-year OS calibration curves in the two groups are shown in Figures 7(a)–7(f). The calibration curves showed a well-consistent OS between the prediction and reality in the two groups.

## 4. Discussion

CRC has the third highest incidence among cancers [1]. Even though nearly 75% of patients with CRC could be potentially treated by surgery [28], CRC still ranks third in the highest mortality among cancers, and it continues to seriously endanger human health. Therefore, clinicians need to estimate the outcome and decide on subsequent treatment.

The TNM system is a common staging system in the diagnosis and treatment of patients with CRC [5]. This system stages cancer based on three aspects: the degree of cancer invasion, metastasis at regional lymph nodes, and the invasion situation of a distant organ. This system is simple and easy to use, but it still has its shortcomings. Several studies reported that ELNs during surgery could influence the prognosis of patients with CRC [29, 30]. Le Voyer et al. reported that the outcome of patients with ELNs of more than 40 was obviously better than that of patients with ELNs of less than 10 [29]. One explanation is that insufficient ELNs obtained during surgery could directly impair the accuracy of tumor staging [29], thus influencing the choice of subsequent treatment options. A study reported that the more ELNs obtained, the better the prognosis of patients with CRC, and at least 20 ELNs should be obtained during surgery [31]. Guidelines also recommended that at least 12 ELNs should be obtained during surgery [13]. However, the AJCC TNM staging system does not take ELNs into consideration. Thus, LNR and LODDS should be introduced [14, 32, 33].

LNR is the proportion of PLNs that make up ELNs and has been reported as a good predictor of outcomes in several kinds of cancer [15–18]. One study that focused on right colon cancer pointed out that LNR is a potentially valuable factor in predicting the probability of tumor recurrence [15]. However, LNR also has its inherent shortcomings. When the number of PLNs is 0, the value of LNR does not change regardless of the number of ELNs. LODDS, which refers to the logarithm of the result of PLNs divided by NLNs, showed better prediction ability than LNR in several studies [19–21]. Even when the PLN is 0, LODDS could differentiate patients in accordance with different ELNs. A research study reported that LODDS could be a potential factor in predicting the outcome of patients with CRC [34]. Arslan et al. further indicated that the LODDS classification showed better prediction ability in patients with ELNs less than 12 during surgery [35]. Additional studies are needed to explore which one is better.

A total of 14,039 cases of histological grades III-IV CRC were downloaded from the SEER database and randomly divided into two groups for model construction and validation. LODDS was identified in the logistic and Cox regression analyses. Meanwhile, LNR and the AJCC N staging system did not show a significant association with patient OS. Finally, a nomogram was created to visualize the results. This nomogram was built on the basis of LODDS, and it showed well prediction efficiency. This result is consistent with previous discussions.

This study has limitations. First, the cutoff value of LODDS was decided on the basis of its tertiles, as in the research conducted by Lee et al. [25]. Therefore, an optimal cutoff value should be further explored through follow-up studies to improve the reliability of this predictive model. Second, all cases involved in this study were downloaded from the SEER database. Cases from additional sources must be verified to improve the accuracy of the model.

## 5. Conclusion

LODDS was found to be a valuable predictive factor, and it showed better predictive ability for the OS of patients with histological grades III-IV CRC than LNR. Race, AJCC T stage, AJCC M stage, LODDS,histological type, bone metastasis, and liver metastasis were selected as isolated factors to construct a nomogram. The nomogram performed well in both groups. All variables involved in the nomogram were easily obtained in the clinical diagnosis and treatment of patients with CRC. The nomogram could provide a certain reference for doctors to assess the outcome of patients with histological grades III-IV CRC and choose subsequent treatment.

## Figures and Tables

**Table 1:** Demographics and clinical characteristics of patients in the training and validation group.

Variates	All patients (*n* = 14039)	Group		*p* value
		Training dataset (*n* = 9828)	Validation dataset (*n* = 4211)	
Age, no. (%)				
<45 years old	1423 (10.1)	975 (9.9)	448 (10.6)	0.192
45–60 years old	4777 (34.0)	3384 (34.4)	1393 (33.1)	
>60 years old	7839 (55.8)	5469 (55.6)	2370 (56.3)	
Sex, no. (%)				
Male	7167 (51.1)	5004 (50.9)	2163 (51.4)	0.638
Female	6872 (48.9)	4824 (49.1)	2048 (48.6)	
Race, no. (%)				
White	11219 (79.9)	7854 (79.9)	3365 (79.9)	0.941
Black	1566 (11.2)	1092 (11.1)	474 (11.3)	
Other	1254 (8.9)	882 (9.0)	372 (8.8)	
AJCC stage, no. (%)				
Stage I	1335 (9.5)	952 (9.7)	383 (9.1)	0.659
Stage II	3148 (22.4)	2187 (22.3)	961 (22.8)	
Stage III	6278 (44.7)	4388 (44.6)	1890 (44.9)	
Stage IV	3278 (23.3)	2301 (23.4)	977 (23.2)	
AJCC T stage, no. (%)				
T1	790 (5.6)	557 (5.7)	233 (5.5)	0.569
T2	1204 (8.6)	838 (8.5)	366 (8.7)	
T3	7775 (55.4)	5476 (55.7)	2299 (54.6)	
T4	4270 (30.4)	2957 (30.1)	1313 (31.2)	
AJCC N stage, no. (%)				
N0	4767 (34.0)	3335 (33.9)	1432 (34.0)	0.646
N1	4339 (30.9)	3059 (31.1)	1280 (30.4)	
N2	4933 (35.1)	3434 (34.9)	1499 (35.6)	
AJCC M stage, no. (%)				
M0	10761 (76.7)	7527 (76.6)	3234 (76.8)	0.803
M1	3278 (23.3)	2301 (23.4)	977 (23.2)	
Grade, no. (%)				
Grade III	11721 (84.5)	8198 (83.4)	3523 (83.7)	0.736
Grade IV	2318 (16.5)	1630 (16.6)	688 (16.3)	
Histological type, no. (%)				
Adenocarcinoma	12112 (86.3)	8480 (86.3)	3632 (86.3)	0.773
AM/MPA	1282 (9.1)	890 (9.1)	392 (9.3)	
SRCC	645 (4.6)	458 (4.7)	187 (4.4)	
Primary site, no. (%)				
Left colon	3495 (24.9)	2445 (24.9)	1050 (24.9)	0.551
Right colon	7645 (54.5)	5376 (54.7)	2269 (53.9)	
Rectum	2899 (20.6)	2007 (20.4)	892 (21.2)	
LNR, no. (%)				
LNR ≤ 0.24	9507 (67.7)	6671 (67.9)	2866 (67.9)	0.551
LNR > 0.24	4532 (32.3)	3157 (32.1)	1375 (32.7)	
LODDS, no. (%)				
LODDS1	4761 (33.9)	3318 (33.8)	1443 (34.3)	0.509
LODDS2	4690 (33.4)	3313 (33.7)	1377 (32.7)	
LODDS3	4588 (32.7)	3197 (32.5)	1391 (33.0)	
Examined lymph nodes, no. (%)				
Low	1476 (10.5)	1016 (10.3)	460 (10.9)	0.314
High	12563 (89.5)	8812 (89.7)	3751 (89.1)	
Negative lymph nodes, no. (%)				
Low	3229 (23.0)	2231 (22.7)	998 (23.7)	0.205
High	10810 (77.0)	7597 (77.3)	3213 (76.3)	
Metastasis at bone, no. (%)				
Yes	101 (0.7)	70 (0.7)	31 (0.7)	0.964
No	13938 (99.3)	9758 (99.3)	4180 (99.3)	
Metastasis at brain, no. (%)				
Yes	35 (0.2)	28 (0.3)	7 (0.2)	0.268
No	14004 (99.8)	9800 (99.7)	4204 (99.8)	
Metastasis at liver, no. (%)				
Yes	2086 (14.9)	1456 (14.8)	630 (15.0)	0.844
No	11953 (85.1)	8372 (85.2)	3581 (85.0)	
Metastasis at lung, no. (%)				
Yes	426 (3.0)	302 (3.1)	124 (2.9)	0.725
No	13913 (97.0)	9526 (96.9)	4087 (97.1)	
Survival status, no. (%)				
Dead	5418 (38.6)	3813 (38.8)	1605 (38.1)	0.458
Alive	8621 (61.45)	6015 (61.2)	2606 (61.9)	
Survival time (month)	33.28 (22.82)	33.49 (22.95)	32.80 (22.51)	0.103
Mean (SD)				

AJCC, American Joint Committee on Cancer; T, tumor; N, nodes; M, metastasis; LNR, lymph node ratio; LODDS, the log odds of positive lymph nodes; AM/MPA, mucinous adenocarcinoma\mucin-producing adenocarcinoma; SRCC, signet ring cell carcinoma; SD, standard deviation.

**Table 2:** The univariate and multivariate logistic regression analysis of overall survival in the training cohort.

Variates	Univariate logistic regression analysis				LASSO-logistic regression analysis			
	OR	2.5% CI	97.5% CI	*P* value	OR	2.5% CI	97.5% CI	*P* value
Age								
<45 years old	Reference				Reference			
45–60 years old	1.070	0.921	1.240	0.385	1.222	1.022	1.463	0.029
>60 years old	1.290	1.120	1.490	<0.001	1.994	1.677	2.375	<0.001
Sex								
Male	Reference				Reference			
Female	0.890	0.820	0.965	0.005	0.859	0.778	0.948	0.003
Race								
White	Reference				Reference			
Black	1.230	1.080	1.400	0.002	1.242	1.063	1.451	0.006
Other	0.904	0.781	1.050	0.173	0.926	0.776	1.103	0.389
Primary site								
Left colon	Reference				Reference			
Right colon	1.180	1.070	1.300	0.001	1.302	1.152	1.473	<0.001
Rectum	0.812	0.717	0.918	0.001	1.119	0.964	1.300	0.140
Pathology grade								
Grade III	Reference							
Grade IV	1.270	1.140	1.410	<0.001				
AJCC T stage								
T1	Reference				Reference			
T2	1.540	1.100	2.140	0.012	1.403	0.993	2.002	0.058
T3	4.420	3.350	5.840	<0.001	2.391	1.789	3.251	<0.001
T4	14.100	10.700	18.700	<0.001	5.145	3.821	7.046	<0.001
AJCC N stage								
N0	Reference				Reference			
N1	2.630	2.350	2.950	<0.001	0.963	0.788	1.176	0.714
N2	6.720	6.020	7.510	<0.001	1.018	0.796	1.302	0.884
AJCC M stage								
M0	Reference				Reference			
M1	9.470	8.470	10.600	<0.001	3.674	3.068	4.412	<0.001
Histological type								
Adenocarcinoma	Reference				Reference			
AM/MPA	1.510	1.310	1.730	<0.001	1.214	1.029	1.432	0.021
SRCC	2.220	1.830	2.690	<0.001	1.637	1.301	2.061	<0.001
LNR								
Low	Reference				Reference			
High	5.470	4.990	6.000	<0.001	1.174	0.609	2.241	0.629
LODDS								
LODDS1	Reference				Reference			
LODDS2	2.630	2.340	2.950	<0.001	1.897	1.553	2.32	<0.001
LODDS3	9.400	8.370	10.600	<0.001	3.092	1.587	6.078	0.001
Examined lymph nodes								
Low	Reference							
High	0.563	0.494	0.641	<0.001				
Negative lymph nodes								
Low	Reference				Reference			
High	0.225	0.203	0.248	<0.001	0.583	0.505	0.674	<0.001
Metastasis at bone								
No	Reference				Reference			
Yes	38.700	12.200	123.000	<0.001	5.852	2.110	24.349	0.003
Metastasis at brain								
No	Reference							
Yes	6.370	2.600	15.600	<0.001				
Metastasis at liver								
No	Reference							
Yes	8.430	7.350	9.670	<0.001				
Metastasis at lung								
No	Reference				Reference			
Yes	6.880	5.100	9.290	<0.001	1.794	1.439	2.234	<0.001

AJCC, American Joint Committee on Cancer; T, tumor; N, nodes; M, metastasis; LNR, Lymph node ratio; AM/MPA, mucinous adenocarcinoma\mucin-producing adenocarcinoma; SRCC, signet ring cell carcinoma; OR, odds ratio; CI, confidence interval.

**Table 3:** The univariate and multivariate Cox regression analysis of overall survival in the training cohort.

Variates	Univariate Cox				Multivariate Cox			
	HR	95% CI		*P* value	HR	95% CI		*P* value
Age								
<45 years old	Reference							
45–60 years old	1.060	0.942	1.190	0.330				
>60 years old	1.230	1.100	1.370	<0.001				
Sex								
Male	Reference							
Female	0.903	0.848	0.963	0.002				
Race								
White	Reference				Reference			
Black	1.140	1.030	1.260	0.010	1.126	1.020	1.242	0.018
Other	0.941	0.839	1.050	0.295	0.839	0.749	0.941	0.003
Primary site								
Left colon	Reference							
Right colon	1.160	1.070	1.250	<0.001				
Rectum	0.845	0.765	0.933	0.001				
AJCC T stage								
T1	Reference				Reference			
T2	1.140	0.844	1.530	0.399	1.083	0.804	1.459	0.599
T3	3.090	2.420	3.930	<0.001	1.839	1.441	2.348	<0.001
T4	7.630	5.990	9.720	<0.001	3.031	2.367	3.88	<0.001
AJCC M stage								
M0	Reference				Reference			
M1	5.300	4.960	5.660	<0.001	2.464	2.236	2.715	<0.001
Histological type								
Adenocarcinoma	Reference				Reference			
AM/MPA	1.310	1.180	1.450	<0.001	1.077	0.971	1.195	0.159
SRCC	1.950	1.720	2.210	<0.001	1.498	1.315	1.707	<0.001
NLNs								
Low	Reference							
High	0.316	0.296	0.338	<0.001				
LODDS								
LODDS1	Reference				Reference			
LODDS2	2.250	2.030	2.490	<0.001	1.719	1.551	1.905	<0.001
LODDS3	6.260	5.700	6.870	<0.001	3.402	3.077	3.761	<0.001
Metastasis at liver								
No	Reference				Reference			
Yes	4.440	4.140	4.760	<0.001	1.366	1.236	1.510	<0.001
Metastasis at bone								
No	Reference				Reference			
Yes	7.030	5.520	8.970	<0.001	2.253	1.763	2.880	<0.001

AJCC, American Joint Committee on Cancer; T, tumor; M, metastasis; LODDS, the log odds of positive lymph nodes; AM/MPA, mucinous adenocarcinoma\mucin-producing adenocarcinoma; SRCC, signet ring cell carcinoma; NLN, negative regional lymph nodes; HR, hazard ratio; CI, confidence interval.

## Data Availability

The primary data used to support the findings of this study are available from the corresponding author upon request.
